# Digital Assessment of Cognitive Health in Outpatient Primary Care: Usability Study

**DOI:** 10.2196/66695

**Published:** 2025-03-12

**Authors:** Adam J Doerr, Taylor A Orwig, Matthew McNulty, Stephanie Denise M Sison, David R Paquette, Robert Leung, Huitong Ding, Stephen B Erban, Bruce R Weinstein, Yurima Guilarte-Walker, Adrian H Zai, Allan J Walkey, Apurv Soni, David D McManus, Honghuang Lin

**Affiliations:** 1 Program in Digital Medicine Department of Medicine University of Massachusetts Chan Medical School Worcester, MA United States; 2 Division of General Internal Medicine Department of Medicine University of Massachusetts Chan Medical School Worcester, MA United States; 3 Massachusetts AI and Technology Center Manning College of Information and Computer Sciences University of Massachusetts Amherst Amherst, MA United States; 4 Department of Anatomy and Neurobiology Chobanian & Avedisian School of Medicine Boston University Boston, MA United States; 5 Division of Health System Science Department of Medicine University of Massachusetts Chan Medical School Worcester, MA United States; 6 Division of Health Informatics and Implementation Science Department of Population and Quantitative Health Sciences University of Massachusetts Chan Medical School Worcester, MA United States

**Keywords:** cognitive assessment, primary care, digital, cognitive impairment, digital assessment, assessment, cognitive health, cognition, primary care, cognitive evaluation, Core Cognitive Evaluation, CCE, cohort, impairment, cognitive, outpatient

## Abstract

**Background:**

Screening for cognitive impairment in primary care is important, yet primary care physicians (PCPs) report conducting routine cognitive assessments for less than half of patients older than 60 years of age. Linus Health’s Core Cognitive Evaluation (CCE), a tablet-based digital cognitive assessment, has been used for the detection of cognitive impairment, but its application in primary care is not yet studied.

**Objective:**

This study aimed to explore the integration of CCE implementation in a primary care setting.

**Methods:**

A cohort of participants was recruited from the upcoming schedules of participating PCPs at UMass Memorial Medical Center. Eligibility criteria included individuals aged ≥65 years; ability to read, write, and speak in English or Spanish; no previous diagnosis of cognitive impairment; and no known untreated hearing or vision impairment. Research coordinators collected consent from participants and facilitated the screening process. PCPs reviewed reports in real time, immediately before the scheduled visits, and shared results at their discretion. A report was uploaded to each participant’s REDCap (Research Electronic Data Capture; Vanderbilt University) record and linked to the encounter in the electronic health record. Feedback from patients and their caregivers (if applicable) was collected by a tablet-based survey in the clinic before and after screening. Participating PCPs were interviewed following the completion of the study.

**Results:**

The screened cohort included 150 patients with a mean age of 74 (SD 7) years, of whom 65% (97/150) were female. The CCE identified 40 patients as borderline and 7 as positive for cognitive impairment. A total of 84 orders were placed for select laboratory tests or referrals to neurology and neuropsychology within 20 days of CCE administration. Before the assessment, 95% (143/150) of patients and all 15 caregivers expressed a desire to know if their or their loved one’s brain health was declining. All except one patient also completed the postassessment survey. Among them, 96% (143/149) of patients reported finding the CCE easy to complete, and 70% (105/149) felt that the experience was beneficial. In addition, 87% (130/149) of patients agreed or strongly agreed that they wanted to know their CCE results. Among the 7 participating PCPs, 6 stated that the CCE results influenced their patient care management, and all 7 indicated they would continue using the CCE if it were made available after the study.

**Conclusions:**

We explored the integration of the CCE into primary care visits, which showed minimal disruption to the practice workflow. Future studies will be warranted to further validate the implementation of digital cognitive impairment screening tools within primary care settings in the real world.

## Introduction

Dementia is a neurodegenerative disorder characterized by progressive deficits in one or more cognitive domains severe enough to interfere with an individual’s functional status [1]. Mild cognitive impairment (MCI) is a precursor of dementia, characterized by more subtle cognitive deficits that do not interfere with functional status [1]. Although definitional variability and reporting bias complicate the estimation of prevalence [2], MCI likely afflicts 12% to 18% of people aged 60 years and older [3,4]. The rate of progression from MCI to dementia is estimated to be 10% to 15% yearly [5].

Only about 18% of Americans are familiar with MCI [1]. However, when MCI is described, 85% report they would want to know if they had either MCI or early dementia, with reasons cited including to plan for the future, to enable earlier treatment of symptoms, to take steps to prevent further deterioration, and to better understand their condition [1]. Similar sentiments are held by family members, for whom early detection enables a more gradual adaptation to the caregiver role, resulting in an improved sense of competence and less psychological distress [6,7]. On a broader level, early detection can delay institutionalization and inform the allocation of human resources within a health care system already marred by shortages in the dementia care workforce [8-10].

Most primary care physicians (PCPs) believe screening for MCI and dementia in older patients is important [1]. Following the detection of MCI or early dementia, PCPs commonly intervene by assessing for reversible causes, recommending lifestyle changes, and referring to specialists [1]. However, most PCPs feel uncomfortable in this domain and report conducting cognitive assessments for less than half of patients older than 60 years of age [1]. Reported barriers to screening in primary care settings include low provider confidence (particularly in distinguishing MCI from normal aging), time constraints, competing priorities, poorly defined protocols for screening, and lack of financial incentives [1,11,12]. Only 40% of PCPs report being familiar with existing cerebrospinal fluid and imaging biomarker tests that can aid in the diagnosis of dementia, while only 18% of patients are referred for such testing when MCI is detected [1,11,12]. An unfortunate consequence is that more than 90% of patients with MCI are not identified [13], resulting in delayed diagnosis when advanced stages of dementia have developed, disability is higher, treatment options are limited [14], and caregiver stress is high [15].

Alzheimer's disease is the most common neurodegenerative cause of MCI and dementia in individuals aged ≥65 years. Biomarkers for AD span multiple modalities, including molecular profiling of bodily fluids, brain imaging, electroencephalography, and neuropsychological testing [16]. With increased computational power, particularly in the form of artificial intelligence, digital biomarkers have emerged as promising new noninvasive approaches for early detection of MCI and early dementia due to AD [17]. One example is the digitization of the traditional clock drawing test (CDT). Although thoroughly validated, the CDT is a classically analog test that requires manual supervision and skilled interpretation, limiting its use in busy clinical practice and making it susceptible to interobserver variability [18]. Linus Health’s Core Cognitive Evaluation (CCE) is a tablet-based cognitive assessment tool consisting of an immediate recall task, a digital CDT administered on an Apple iPad using an Apple Pencil, and a delayed recall task (collectively termed Digital Clock and Recall [DCR]) [19,20]. Performance is analyzed by a machine learning algorithm to yield a score (ranging from 0 to 5), which is indicative of cognitive impairment (0-1), borderline for cognitive impairment (2-3), or not indicative of cognitive impairment (4-5). The DCR is paired with a Life and Health Questionnaire (LHQ), which screens for modifiable lifestyle and psychosocial risk and protective factors related to brain health, enabling prediction of future dementia risk and personalized recommendations for patients. The DCR has demonstrated greater sensitivity and accuracy than existing cognitive assessment tests such as the Mini-Mental State Examination (MMSE) and the Mini-Cog [21] and takes substantially less time to complete (approximately 3 min).

These qualities make the CCE well suited for the busy primary care setting. However, the impact of the CCE in primary care with respect to patient and provider satisfaction, downstream clinical decision-making, and reimbursement remains unclear. The objective of this study was to evaluate the feasibility and impact of screening for cognitive impairment in an outpatient, primary care setting using the Linus Health CCE.

## Methods

### Screening and Recruitment

Participants for this study were recruited from the Benedict Adult Primary Care Clinic at UMass Memorial Medical Center. Eligible patients were identified through a list of upcoming visits generated from the electronic health record system (Epic Systems). Eligibility criteria included being aged 65 years or older; having the ability to read, write, and speak in English or Spanish; having no previous diagnosis of cognitive impairment; and having no untreated hearing or vision impairment that would hinder audible conversation or the use of a tablet. Recruitment letters were mailed to eligible patients, inviting them to participate in the study. These letters included an opt-out phone number and email address. If no response was received, a research coordinator followed up with a phone call 3-5 days after the letter was sent to assess interest. Patients who expressed interest received another phone call one day before their scheduled visit to confirm their participation, remind them to arrive an hour early, and advise them to bring their reading glasses or hearing aids, if applicable. All contacted patients were logged in a recruitment tracker, which recorded details such as patient name, participation status, PCP name, recruitment letter mail date, follow-up call date, visit date and type, and participation reminder call date.

### Assessment

Participants were greeted in the clinic by research coordinators, escorted to a quiet and private room without an analog clock present, and completed the consent process. They then completed preassessment surveys on study tablets. If the patients were accompanied by their caregivers, their caregivers were also provided with a fact sheet and completed a survey. Simultaneously, research coordinators registered participants on a separate tablet equipped with the Linus Health CCE. Participants then completed the CCE on a study tablet. Participants were first administered the DCR, in the following sequence: (1) during the immediate recall condition, 3 words were voiced by the tablet, and the patient was asked to repeat them immediately; (2) during the command clock condition, the tablet voiced the following command: “draw the face of a clock, put in all of the numbers, and set the hands to ‘10 after 11”’; (3) during the copy clock condition, the tablet displayed a picture of an analog clock set to 11:10 and asked the patient to copy it; and (4) during the delayed recall condition, the patient was asked to repeat the 3 words initially voiced by the tablet. The DCR was not adjusted for demographics or education similar to the original Mini-Cog score. Patients then completed the LHQ and a postassessment survey on the study tablet before their visit with the PCP. A total of 7 PCPs who consented to participate were identified and given a fact sheet. They went through a training session before the study. After assessments were completed for the entire cohort, all PCPs involved in the study completed a provider feedback survey sent by email and completed an interview assessing their impressions of the CCE.

### Results Sharing

After completion of the CCE, results were immediately uploaded into the Linus Health portal. This generated a provider-facing report consisting of DCR scores; interpretations; playbacks of clock drawing and verbal recall; clinical decision support; and a personalized patient-facing Brain Health Action Plan, which identifies modifiable risk factors for cognitive impairment and provides evidence-based suggestions for intervention. These results were printed and given to PCPs immediately before patient visits for review in real time. DCR results were shared with patients at the discretion of PCPs, while the Brain Health Action Plans were printed and universally shared with each patient.

### Data Management and Analysis

Study data were collected and managed using REDCap (Research Electronic Data Capture; Vanderbilt University) tools hosted at UMass Chan Medical School [22,23]. Informed consent was also obtained using REDCap within the eConsent framework on a study tablet [24]. REDCap is a secure, web-based software platform designed to support data capture for research studies, providing (1) an intuitive interface for validated data capture; (2) audit trails for tracking data manipulation and export procedures; (3) automated export procedures for seamless data downloads to common statistical packages; and (4) procedures for data integration and interoperability with external sources. Identifiable patient health information was concealed from Linus Health throughout the study and thereafter.

### Ethical Considerations

The study protocol was approved by the Western-Copernicus Group Institutional Review Board (approval number 1341324), and informed consent was obtained from all patients. Caregivers and PCPs were provided with a fact sheet to guide them in completing the survey. No compensation for participation was provided. For data analysis, only deidentified data were exported from REDCap to ensure confidentiality and compliance with ethical standards.

## Results

### Screened Cohort

A flowchart of participant recruitment is shown in Figure S1 in Multimedia Appendix 1. During the screening process, 1469 patients were identified as meeting the eligibility criteria. Among them, 160 accepted the invitation to participate. Of these, 9 either did not show up, changed their minds, or canceled their appointments, and one participant was found ineligible to provide consent. Ultimately, 150 patients from the panels of 7 PCPs were successfully enrolled and completed the CCE between March and December 2023. The baseline demographic and clinical characteristics of studied patients are listed in Table 1. The participants had a mean age of 74 (SD 7) years; 65% (97/150) were female, and 74% (111/150) held a college degree or higher. The CCE identified 40 patients as borderline and 7 patients as positive for cognitive impairment. The number of patients recruited from each PCP panel ranged from 9 to 38.

**Table 1:** Patient demographic and clinical characteristics. Mean (SD) values for continuous variables, and counts (%) for categorical variables have been reported.

Characteristics			Values (n=150)
**Age (years), mean (SD)**			74 (7)
**Female, n (%)**			97 (65)
**Race, n (%)**			
	White	147 (98)	
	Black	2 (1)	
	Not reported or unknown	1 (1)	
**Ethnicity, n (%)**			
	Hispanic or Latino	2 (1)	
	Not Hispanic or Latino	136 (91)	
	Not recorded or unknown	12 (8)	
**Education, n (%)**			
	High school	11 (7)	
	Some college	28 (19)	
	College graduate and above	111 (74)	
**Hypertension, n (%)**			111 (74)
**Diabetes mellitus, n (%)**			32 (21)
**Hyperlipidemia, n (%)**			135 (90)
**Smoking, n (%)**			35 (23)
**Ischemic heart disease, n (%)**			27 (18)
**Atrial fibrillation or flutter, n (%)**			24 (16)
**Heart failure, n (%)**			10 (7)
**Peripheral vascular disease, n (%)**			7 (5)
**Obstructive sleep apnea, n (%)**			62 (41)
**Parkinson disease, n (%)**			1 (1)
**Depression, n (%)**			37 (25)
**Stroke, n (%)**			9 (6)
**Transient ischemic attack, n (%)**			10 (7)
**Epilepsy, n (%)**			4 (3)
**Hearing loss, n (%)**			66 (44)
**B_12_ deficiency, n (%)**			3 (2)

### Provider Response to the CCE

Among the 7 PCPs participating in the study, 5 had more than 10 years of clinical practice, one had 5-10 years of clinical practice, and one was a junior clinician. All PCPs stated that it was important to detect undiagnosed cognitive impairment, and most noted time or workflow constraints as a barrier to cognitive testing. All PCPs reviewed test results before meeting with their patient, with an average reported review time of 2 minutes, and more time is needed if the results indicated a cognitive issue. A majority of the PCPs stated that the CCE did not interfere with patient visits (5/7, 71%) and that it did not lead to any stress or challenges for their patient (6/7, 86%). Most PCPs (4/7, 57%) reported that the CCE did not cause them any stresses or challenges; among the 3 providers who answered affirmatively, one discussed the stress of breaking the news of poor CCE scores to patients, another cited the challenge of finding the time to discuss results, and the last voiced concerns about false positive scores that might result from extraneous factors like anxiety, comorbidities, and language barriers. All PCPs stated that they generally agreed with the CCE results, and a majority (6/7, 86%) of PCPs stated that the results of the CCE changed their patient care management. All PCPs reported they would continue using the CCE if made available following the study.

### Downstream Clinical Action Following the CCE

Referrals to neurology and neuropsychology, select laboratory tests possibly related to the workup of dementia, and neuroimaging studies ordered after administration of the CCE are reported in Table 2. There were 84 such orders placed within 20 days of CCE administration, and an additional 58 orders were placed within the subsequent 160 days thereafter. Moreover, among the 7 patients with DCR scores of 0 and 1, a total of 4 (57%) were later diagnosed with cognitive impairment. In contrast, only 16 (11%) of the 143 patients with scores of 2 or above were later diagnosed with cognitive impairment.

**Table 2:** Near-term and delayed cognition-relevant orders placed following the administration of the Core Cognitive Evaluation (CCE).

Cognition-relevant orders		Days since the CCE	
		0-20	21-180
**Referral, n**			
	Neurology	3	2
	Neuropsychology	0	1
**Imaging test, n**			
	CT^a^ head	2	5
	MRI^b^ brain	4	11
	PET^c^ brain	0	2
**Laboratory test, n**			
	B_12_	26	7
	Folate	11	4
	Rapid plasma reagin	3	0
	Thyroid-stimulating hormone	35	26
**Total tests, n**		84	58

^a^CT: computed tomography.

^b^MRI: magnetic resonance imaging.

^c^PET: positron emission tomography.

### Patient and Caregiver Perspectives on Brain Health Prior to the CCE

The results of the preassessment survey for patients and caregivers are displayed in Table 3, and the stratified results are shown in Table S1 in Multimedia Appendix 1. Before the screening, 58% (87/150) of patients agreed or strongly agreed they worry about their brain health, while 95% (143/150) of patients and all caregivers reported they would want to know if their or their loved one’s brain health was declining. Approximately two-thirds of both patients (100/150, 67%) and caregivers (10/15, 67%) agreed or strongly agreed their plans for the next 5-10 years would change in response to a decline in brain health; “How we spend our time” was the most frequently selected decision that would be impacted. About two-thirds (95/150, 63%) of patients reported they go to their PCPs for help with taking care of their brain health.

**Table 3:** Patient and caregiver perspectives on brain health before the administration of the Core Cognitive Evaluation.

Survey questions			Strongly agree, n (%)		Agree, n (%)		Neither agree nor disagree, n (%)		Disagree, n (%)		Strongly disagree, n (%)
**Patients (n=150)**											
	I worry about my memory or brain health.	15 (10)		72 (48)		28 (19)		17 (11)		18 (12)	
	I understand how to take care of my brain health.	48 (32)		69 (46)		29 (19)		3 (2)		1 (1)	
	I go to my primary care physician to help me take care of my brain health.	45 (30)		50 (33)		43 (29)		9 (6)		3 (2)	
	If my brain health was declining, I would want to know.	81 (54)		62 (41)		3 (2)		1 (1)		3 (2)	
	If I found out that my brain health was declining, it would change my plans for the next 5-10 years.	37 (25)		63 (42)		28 (19)		14 (9)		8 (5)	
**Caregivers (n=15)**											
	I worry about my relative’s memory or brain health.	3 (20)		5 (33)		4 (27)		2 (13)		1 (7)	
	I understand how to help my relatives take care of their brain health.	5 (33)		4 (27)		6 (40)		0 (0)		0 (0)	
	I would want to take my relative to their primary care physician to help me take care of their brain health.	7 (47)		6 (40)		1 (7)		1 (7)		0 (0)	
	If my relative’s brain health was declining, I would want to know.	8 (53)		7 (47)		0 (0)		0 (0)		0 (0)	
	If I knew my relative’s brain health was declining, it would change our plans for the next 5-10 years.	4 (27)		6 (40)		4 (27)		0 (0)		1 (7)	

### Patient Satisfaction With the Core Cognitive Evaluation

The results of the postassessment survey for patients are displayed in Table 4, and the stratified results are shown in Table S2 in Multimedia Appendix 1. One patient did not complete the assessment. Among participants who completed the postassessment survey, 96% (143/149) of patients reported being able to easily complete the CCE, while 70% (105/149) reported that completing the CCE was beneficial. Among those who felt the CCE was beneficial, all felt it enabled them to be productive about their brain health, about two-thirds (68/105, 65%) felt it gave them more control over their long-term brain health, and about one-third (37/105, 35%) felt it addressed a current specific concern about their brain health. As a result of taking the CCE, only one patient reported feeling sad or depressed, while 5% (8/149) reported feeling worried or anxious. Most patients (130/149, 87%) agreed or strongly agreed they would like to know their CCE results.

**Table 4:** Patient satisfaction after the administration of the Core Cognitive Evaluation. One participant did not complete the postassessment survey (n=149).

Survey questions	Strongly agree, n (%)	Agree, n (%)	Neither agree nor disagree, n (%)	Disagree, n (%)	Strongly disagree, n (%)
I was able to complete this assessment easily	107 (71)	36 (24)	3 (2)	0 (0)	3 (2)
Taking this assessment was beneficial	46 (31)	59 (39)	35 (23)	6 (4)	3 (2)
Taking this assessment was stressful	0 (0)	8 (5)	16 (11)	44 (29)	81 (54)
This assessment made me feel anxious or worried	0 (0)	8 (5)	12 (8)	39 (26)	90 (60)
This assessment made me feel sad or depressed	0 (0)	1 (1)	6 (4)	32 (21)	110 (73)
I was to know my results from this assessment	84 (56)	46 (31)	8 (5)	3 (2)	8 (5)

## Discussion

### Principal Findings

Cognitive impairment is a formidable public health threat, with significant burdens posed to individual patients, their caregivers, the health care system, and the economy. With two disease-modifying therapies now approved by the US Food and Drug Administration (FDA) [25,26] and commercially available and covered by the Centers for Medicare and Medicaid Services (CMS) [27], early detection of cognitive impairment has assumed new relevance. In this study, a cohort of older outpatients without a previous diagnosis of cognitive impairment was screened in a primary care setting using the CCE.

The preassessment survey overall comported with the existing body of literature suggesting patients are concerned about their brain health and would strongly prefer to detect cognitive impairment as early as possible. Overall, participant satisfaction with the screening process was high, with a low incidence of patient-reported negative psychological impact. A majority of PCPs stated that the CCE results changed their care management, and all PCPs stated they would continue using the CCE if made available following the study.

The protocol used in this study prioritized minimizing disruption in an already busy clinical environment by aligning cognitive assessments with patients’ scheduled appointments. Central to this workflow was a dedicated team member who facilitated consenting, screening, and delivery of results to providers. Although this role was filled by a research coordinator in the study, it could feasibly be assigned to clinical staff, such as medical assistants, in real-world practice given the simplicity of the screening platform and the minimal training required to administer the survey. Meanwhile, the timing of assessments before visits and the provision of concise results with easily digestible interpretations to PCPs obviate the need for manual grading and interpretation, reducing both the time burden and expertise requirements associated with conventional assessments. This design therefore serves as a pragmatic framework for the implementation of a digital cognitive impairment screening program in a real-world primary care setting.

There are noteworthy limitations inherent to this workflow and its evaluation. While potentially more streamlined for PCPs, the study required patients to arrive early for their scheduled appointments. This represents an added medical burden for the older population and may compete with other previsit agenda items. This extra time requirement was necessary to ensure that cognitive assessments could be completed without disrupting the scheduled clinical visits, allowing PCPs to review the results in real time. However, it might also impact a spouse or family member helping the patient to spend more time onsite in the office. This burden could be mitigated in future studies with improved electronic health record integration and automated clinical decision support implementation, or by exploring alternative workflows, such as integrating the CCE into regular appointment times or offering remote assessments. Furthermore, while patients were surveyed before their scheduled visits and were blind to their CCE results, PCPs were surveyed following enrollment completion, introducing the potential for recall bias in their evaluation of the screening process. This retrospective feedback collection allowed PCPs to reflect on their experiences with multiple patients but may have affected the accuracy of their assessments. In addition, the study’s design, which involved a convenience sample from a single primary care clinic, limits the generalizability of the findings. While this approach facilitated close monitoring of the implementation process, it may not fully represent the broader population. Future studies should expand the sample size and include multiple sites across diverse geographic and health care settings. In addition, most of the participants in this study are non-Hispanic White, which greatly limits its generalizability to other racial and ethnic populations. More efforts have to be made to increase the diversity of participants in the future [28]. Finally, irrespective of feasibility, the efficacy of a widely adopted digital cognitive screening program using the CCE remains speculative until tested in a randomized trial. It would be essential to further investigate strategies for sustainable and scalable integration of the CCE in diverse clinical environments, including enhanced electronic health record integration.

### Conclusion

In summary, we explored the integration of the CCE into primary care visits, which showed minimal disruption to the practice workflow. Future studies will be warranted to further validate the implementation of digital cognitive impairment screening tools within primary care settings in the real world.

## Appendix

Multimedia Appendix 1 Flowchart illustrating the participant screening and enrollment, patient perspectives on brain health prior to the administration of the CCE stratified by DCR score groups, and patient satisfaction after the administration of the CCE stratified by DCR score groups. CCE: Core Cognitive Evaluation; DCR: Digital Clock and Recall.

## Abbreviations

CCE: Core Cognitive Evaluation
CDT: clock drawing test
CMS: Centers for Medicare and Medicaid Services
DCR: Digital Clock and Recall
FDA: US Food and Drug Administration
LHQ: Life and Health Questionnaire
MCI: mild cognitive impairment
MMSE: Mini-Mental State Examination
PCP: primary care physicians
REDCap: Research Electronic Data Capture

## Data Availability

The datasets generated or analyzed during this study are available from the corresponding author on reasonable request.
