# Supplementary material for: Digital Assessment of Cognitive Health in Outpatient Primary Care: Usability Study
Source: JMIR Form Res. 2025 Mar 12;9:e66695. doi: 10.2196/66695 (PMC11947626; doi:10.2196/66695)
Supplement: Multimedia Appendix 1 [file formative_v9i1e66695_app1.docx]

**Figure S1**. Flowchart illustrating the participant screening and enrollment.

**
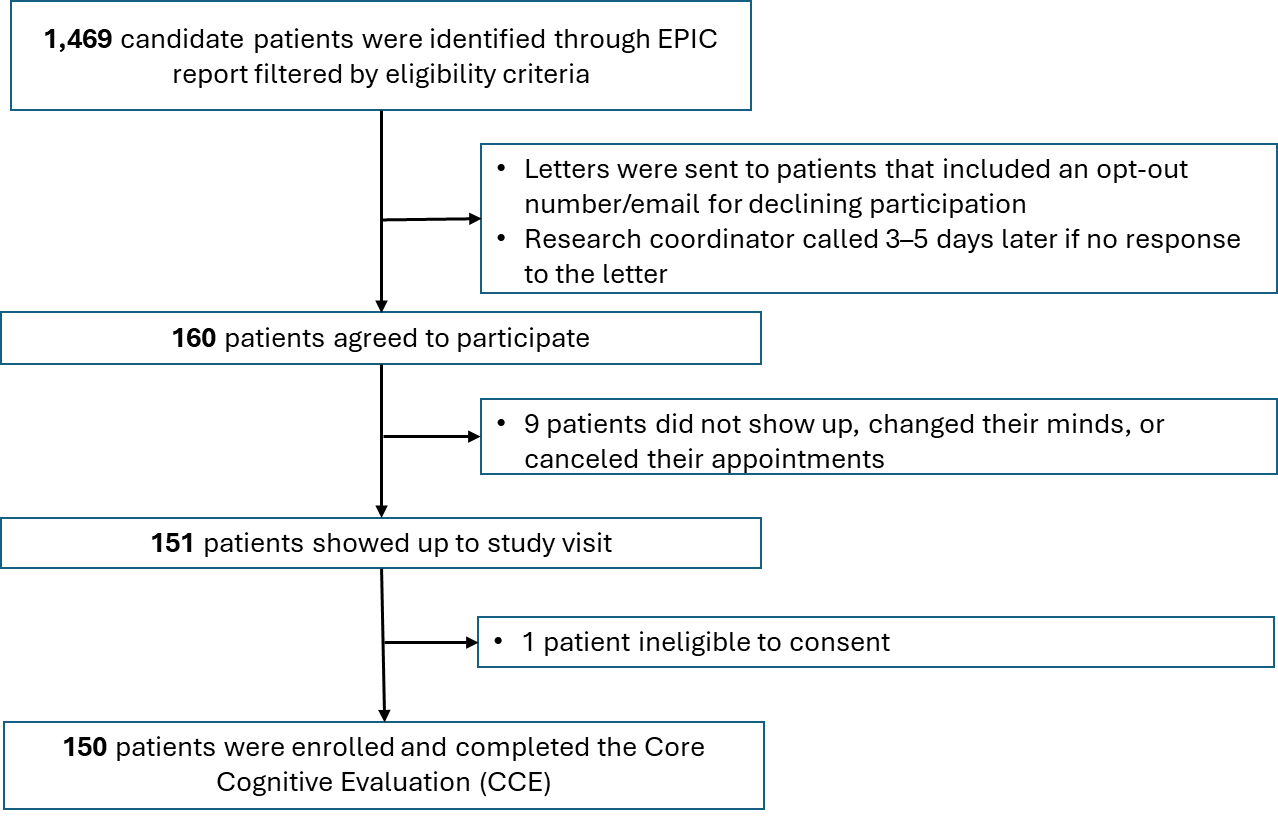
Table S1.** Patient perspectives on brain health prior to the administration of CCE stratified by DCR score groups

|  | Participants with normal scores (N=103) | | | | | Participants with borderline scores (N=40) | | | | | Participants with scores indicating cognitive impairment (N=7) | | | | |
| --- | --- | --- | --- | --- | --- | --- | --- | --- | --- | --- | --- | --- | --- | --- | --- |
|  | Strongly Agree,  N (%) | Agree,  N (%) | Neither Agree nor Disagree, N (%) | Disagree,  N (%) | Strongly Disagree,  N (%) | Strongly Agree,  N (%) | Agree,  N (%) | Neither Agree nor Disagree, N (%) | Disagree,  N (%) | Strongly Disagree,  N (%) | Strongly Agree,  N (%) | Agree,  N (%) | Neither Agree nor Disagree, N (%) | Disagree,  N (%) | Strongly Disagree,  N (%) |
| I worry about my memory or brain health. | 8 (8) | 52 (50) | 20 (19) | 10 (10) | 13 (13) | 6 (15) | 17 (43) | 7 (18) | 6 (15) | 4 (10) | 1 (14) | 3 (43) | 1 (14) | 1 (14) | 1 (14) |
| I understand how to take care of my brain health. | 33 (32) | 51 (50) | 18 (17) | 1 (1) | 0 (0) | 12 (30) | 16 (40) | 9 (23) | 2 (5) | 1 (3) | 3 (43) | 2 (29) | 2 (29) | 0 (0) | 0 (0) |
| I go to my primary care physician to help me take care of my brain health. | 30 (29) | 35 (34) | 30 (29) | 6 (6) | 2 (2) | 13 (33) | 12 (30) | 12 (30) | 3 (8) | 0 (0) | 2 (29) | 3 (43) | 1 (14) | 0 (0) | 1 (14) |
| If my brain health was declining, I would want to know. | 59 (57) | 41 (40) | 3 (3) | 0 (0) | 0 (0) | 20 (50) | 17 (43) | 0 (0) | 1 (3) | 2 (5) | 2 (29) | 4 (57) | 0 (0) | 0 (0) | 1 (14) |
| If I found out that my brain health was declining, it would change my plans for the next 5-10 years | 31 (30) | 42 (41) | 19 (18) | 7 (7) | 4 (4) | 4 (10) | 17 (43) | 8 (20) | 7 (18) | 4 (10) | 2 (29) | 4 (57) | 1 (14) | 0 (0) | 0 (0) |

**Table S2**. Patient satisfaction after the administration of CCE stratified by DCR score groups.

|  | Participants with normal scores (N=102) | | | | | Participants with borderline scores (N=40) | | | | | Participants with scores indicating cognitive impairment (N=7) | | | | |
| --- | --- | --- | --- | --- | --- | --- | --- | --- | --- | --- | --- | --- | --- | --- | --- |
|  | Strongly Agree,  N (%) | Agree,  N (%) | Neither Agree nor Disagree, N (%) | Disagree,  N (%) | Strongly Disagree,  N (%) | Strongly Agree,  N (%) | Agree,  N (%) | Neither Agree nor Disagree, N (%) | Disagree,  N (%) | Strongly Disagree,  N (%) | Strongly Agree,  N (%) | Agree,  N (%) | Neither Agree nor Disagree, N (%) | Disagree,  N (%) | Strongly Disagree,  N (%) |
| I was able to complete this assessment easily | 85 (83) | 16 (16) | 0 (0) | 0 (0) | 1 (1) | 21 (53) | 17 (43) | 1 (3) | 0 (0) | 1 (3) | 1 (14) | 3 (43) | 2 (29) | 0 (0) | 1 (14) |
| Taking this assessment was beneficial | 36 (35) | 38 (37) | 24 (24) | 3 (3) | 1 (1) | 9 (23) | 17 (43) | 11 (28) | 2 (5) | 1 (3) | 1 (14) | 4 (57) | 0 (0) | 1 (14) | 1 (14) |
| Taking this assessment was stressful | 0 (0) | 4 (4) | 7 (7) | 30 (29) | 61 (60) | 0 (0) | 4 (10) | 9 (23) | 11 (28) | 16 (40) | 0 (0) | 0 (0) | 0 (0) | 3 (43) | 4 (57) |
| This assessment made me feel anxious or worried | 0 (0) | 4 (4) | 3 (3) | 27 (26) | 68 (67) | 0 (0) | 4 (10) | 9 (23) | 10 (25) | 17 (43) | 0 (0) | 0 (0) | 0 (0) | 2 (29) | 5 (71) |
| This assessment made me feel sad or depressed | 0 (0) | 0 (0) | 1 (1) | 21 (21) | 80 (78) | 0 (0) | 1 (3) | 5 (13) | 10 (25) | 24 (60) | 0 (0) | 0 (0) | 0 (0) | 1 (14) | 6 (86) |
| I want to know my results from this assessment | 62 (61) | 31 (30) | 4 (4) | 0 (0) | 5 (5) | 19 (48) | 14 (35) | 3 (8) | 2 (5) | 2 (5) | 3 (43) | 1 (14) | 1 (14) | 1 (14) | 1 (14) |

*One participant did not complete the post-assessment survey.
